# Supplementary material for: Splice-Junction-Based Mapping of Alternative Isoforms in the Human Proteome
Source: Cell Rep. Author manuscript; Available in PMC 2020 Jan 15. (PMC6961840; doi:10.1016/j.celrep.2019.11.026)
Supplement: 2 [file NIHMS1546469-supplement-2.zip › DF1/PXD006675/VentricularSeptum/VentricularSeptum_40_NCL_NLVMWILNLLK.pdf]

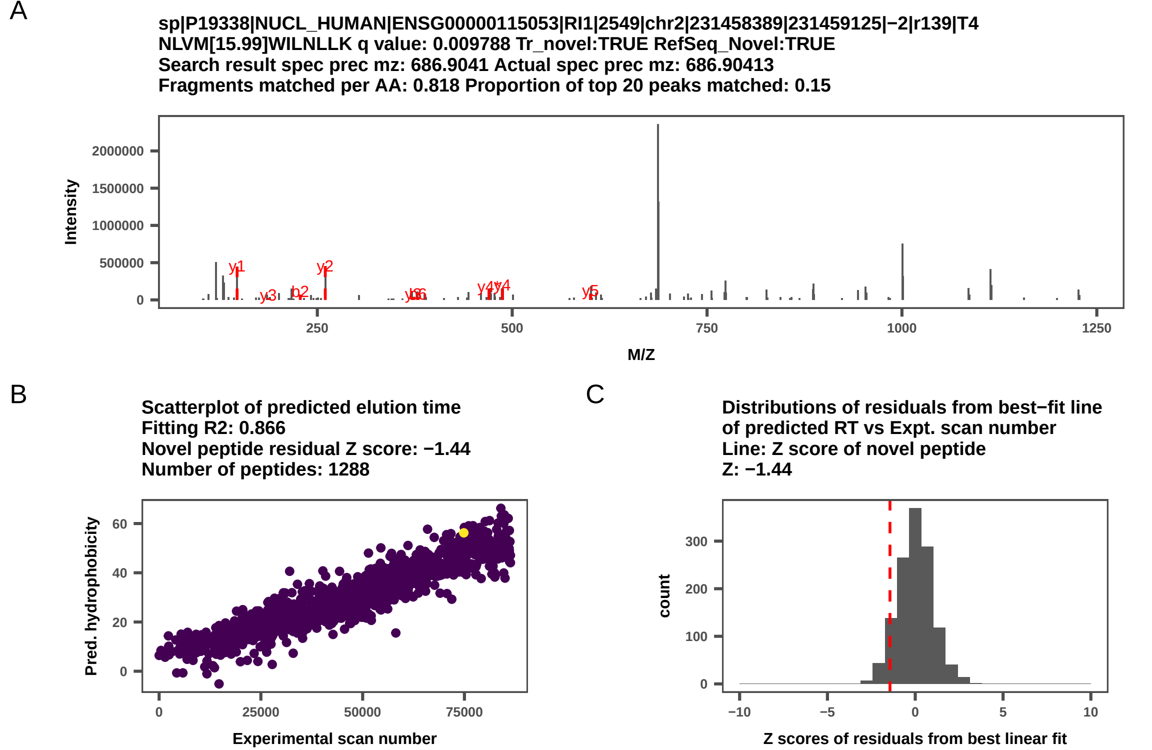
A
sp|P19338|NUCL_HUMAN|ENSG00000115053|RI1|2549|chr2|231458389|231459125|−2|r139|T4
NLVM[15.99]WILNLLK q value: 0.009788 Tr_novel:TRUE RefSeq_Novel:TRUE
Search result spec prec mz: 686.9041 Actual spec prec mz: 686.90413
Fragments matched per AA: 0.818 Proportion of top 20 peaks matched: 0.15
Intensity
2000000
1500000
1000000
500000
0
y1
y2
y3
b2
y4
y5
250
500
750
1000
1250
M/Z
B
Scatterplot of predicted elution time
Fitting R2: 0.866
Novel peptide residual Z score: −1.44
Number of peptides: 1288
Pred. hydrophobicity
60
40
20
0
25000
50000
75000
Experimental scan number
C
Distributions of residuals from best−fit line
of predicted RT vs Expt. scan number
Line: Z score of novel peptide
Z: −1.44
count
300
200
100
0
−10
−5
0
5
10
Z scores of residuals from best linear fit
